# Conscious connected breathing with breath retention intervention in adults with chronic low back pain: protocol for a randomized controlled pilot study

**DOI:** 10.1186/s40814-023-01247-9

**Published:** 2023-01-24

**Authors:** Steven D. Pratscher, Kimberly T. Sibille, Roger B. Fillingim

**Affiliations:** 1 Department of Community Dentistry and Behavioral Science, University of Florida, Gainesville, FL USA; 2 Pain Research and Intervention Center of Excellence, University of Florida, Gainesville, FL USA; 3 Department of Physical Medicine & Rehabilitation, University of Florida, Gainesville, FL USA

**Keywords:** Breathing practice, Breathwork, Conscious connected breathing, Chronic pain, Chronic low back pain, Self-management, Intervention development

## Abstract

**Background:**

Chronic pain is a major source of human suffering, and chronic low back pain (cLBP) is among the most prevalent, costly, and disabling of pain conditions. Due to the significant personal and societal burden and the complex and recurring nature of cLBP, self-management approaches that can be practiced at home are highly relevant to develop and test. The respiratory system is one of the most integrated systems of the body, and breathing is bidirectionally related with stress, emotion, and pain. Thus, the widespread physiological and psychological impact of breathing practices and breathwork interventions hold substantial promise as possible self-management strategies for chronic pain. The primary aim of the current randomized pilot study is to test the feasibility and acceptability of a conscious connected breathing with breath retention intervention compared to a sham control condition.

**Methods:**

The rationale and procedures for testing a 5-day conscious connected breathing with breath retention intervention, compared to a deep breathing sham control intervention, in 24 adults (18–65 years) with cLBP is described. Both interventions will be delivered using standardized audio recordings and practiced over 5 days (two times in-person and three times at-home), and both are described as Breathing and Attention Training to reduce possible expectancy and placebo effects common in pain research. The primary outcomes for this study are feasibility and acceptability. Feasibility will be evaluated by determining rates of participant recruitment, adherence, retention, and study assessment completion, and acceptability will be evaluated by assessing participants’ satisfaction and helpfulness of the intervention. We will also measure other clinical pain, psychological, behavioral, and physiological variables that are planned to be included in a follow-up randomized controlled trial.

**Discussion:**

This will be the first study to examine the effects of a conscious connected breathing with breath retention intervention for individuals with chronic pain. The successful completion of this smaller-scale pilot study will provide data regarding the feasibility and acceptability to conduct a subsequent trial testing the efficacy of this breathing self-management practice for adults with cLBP.

**Trial registration:**

Clinicaltrials.gov, identifier NCT04740710. Registered on 5 February 2021.

**Supplementary Information:**

The online version contains supplementary material available at 10.1186/s40814-023-01247-9.

## Background

Chronic pain is a major public health problem. Chronic low back pain (cLBP), one of the most prevalent chronic pain conditions, affects more than 540 million people and is a leading cause of disability in the world [1, 2]. The significant direct (e.g., healthcare) and indirect costs (e.g., work disability, loss of productivity) associated with cLBP produce substantial personal and societal burden, which is expected to increase over the coming years [1, 3]. Although cLBP tends to resolve over time, there are high rates of recurrence and many people suffer indefinitely [4]. Nonpharmacological interventions, including complementary and integrative approaches, continue to gain interest and are now recommended as first line treatments for cLBP [5, 6]. Due to the persistent, costly, and complex nature of cLBP, nonpharmacological self-management approaches rooted in the biopsychosocial model of chronic pain are of great interest to develop and test [3, 5, 7–16].

Self-management approaches include strategies or interventions that individuals can learn and apply on their own to prevent or relieve symptoms, such as pain or stress [17, 18]. These approaches empower individuals with tools, knowledge, or techniques to take an active role in their health and symptom self-management. This is in contrast to traditional healthcare models where a patient is a passive recipient of care (e.g., surgery). Some examples of self-management approaches for chronic pain include physical exercise, mindfulness meditation, diet, and the use of medical or non-medical devices (e.g., transcutaneous electrical nerve stimulation) [5, 19]. Research shows that people with chronic pain value self-management approaches that (1) they can engage in at-home or remotely, (2) they can apply while continuing their medication, (3) improve more than just pain symptoms (e.g., depression), and (4) have a low time burden [20, 21]. We propose that brief, daily breathing practices are promising self-management approaches to investigate as they align with stakeholder values and potentially offer a nonpharmacological, biopsychosocial, and scalable solution to reduce the global burden of cLBP.

There are a variety of breathwork interventions (i.e., conscious breathing practices) that may be helpful for managing and treating chronic pain [22–26]. For example, increasing evidence supports that slow deep breathing and paced breathing (e.g., respiratory biofeedback) can improve pain-related outcomes and mechanisms, including increased parasympathetic nervous system activity (i.e., heart rate variability), baroflex sensitivity, and relaxation, and decreased stress, anxiety, depression, negative affect, muscle tension, and experimental pain sensitivity [27–38]. These studies, however, primarily examine the effects of controlled breathing practices in healthy samples. Research on breathing practices for those with chronic back pain shows small-to-large effects for improving pain [23–25], but more randomized trials with rigorous methods are needed. Moreover, substantially less research has investigated other types of breathing interventions that may be more potent than slow deep breathing or other similar relaxation-based self-management practices (e.g., meditation) [39]. Specifically, c*onscious connected breathing*, a technique at the core of many different breathwork interventions [40–45], involves breathing with no pause between inhalation and exhalation (also known as circular breathing). Breathwork interventions that use conscious connected breathing often include other “add-on” components, such as mindful body awareness, music, or movement, that are believed to increase their efficacy [46]. The breathing practice investigated in the current study combines conscious connected breathing with periods of breath holding, or breath retention.

The empirical research on conscious connected breathing with breath retention (CCBR) comes from an intervention that is often delivered by a specific teacher (i.e., Wim Hof^1^) within a group retreat setting and combined with additional components (e.g., cold exposure, visualization meditation, strength exercises) [47–50]. These aspects limit accessibility to the intervention and also complicate conclusions that can be drawn regarding the efficacy and mechanisms of the intervention due to the many potential specific and non-specific treatment effects [51–56]. Nevertheless, studies suggest that the breathing and retention practice alone can reduce stress [57] and induce profound physiological changes, including safe respiratory alkalosis (i.e., rise in pH levels), intermittent hypoxia, immediate changes in metabolic and hormonal activity (e.g., increased gluconeogenesis, human growth hormone), and increases in plasma epinephrine levels associated with sympathetic nervous system activation [47–49, 56, 58–60]. CCBR also seems to have significant effects on the immune system, demonstrated by an increase in anti-inflammatory and decrease in pro-inflammatory cytokines following the administration of an endotoxin [48, 56]. Furthermore, the full intervention that included cold exposure and strength exercises was shown safe and feasible for patients with axial spondyloarthritis, a chronic inflammatory disease characterized by pain, with preliminary evidence for reductions in systemic inflammation and disease severity [47]. Yet, CCBR alone has not been tested for its impact on chronic pain, including in those with cLBP.

Consistent with models and recommendations for developing theoretically informed interventions [61, 62], several lines of evidence support the premise that CCBR will be helpful for individuals with chronic pain. First, both the volitional control of breathing and the specific type of rhythmic connected breathing and breath holding technique used in CCBR can elicit a cascade of biological and physiological responses that may reduce pain, such as improvements in heart rate variability (HRV) [26, 63, 64], baroreceptor sensitivity [65–68], acid-base balance (respiratory alkalosis) [69–71], periaqueductal gray structure and function [72–76], and immune and metabolic activity (e.g., inflammation) [47–49, 58, 77, 78]. Second, both the cyclical breathing and the breath retention may serve as a mild stressor (i.e., hormesis), like physical exercise, where the acute challenge to breathe deeper and hold one’s breath for longer than usual results in adaptive changes (e.g., stress resilience, preparation for oxidative stress, carbon dioxide tolerance) [41, 59, 79–84]. Third, conscious breathing exercises are likely to increase mindfulness and interoception (i.e., attention to and awareness of body, feelings, and homeostatic signals) [35, 85, 86]—both considered key factors for self-managing chronic pain as well as for improving overall mental and physical health [87–89]. Last, the extended breath retention (1–2.5 min) may induce intermittent hypoxia, which has been shown to lead to improved respiratory and non-respiratory motor function and neuroplasticity [90–92]. Enhanced neuroplasticity may open a therapeutic window that when combined with existing pain treatments, such as relaxation or mindful body awareness, can have synergistic effects in improving pain-related outcomes [92–97]. While this list is far from exhaustive, these possible mechanistic pathways provide compelling justification to scientifically investigate the physical and psychological effects of CCBR in those with chronic pain.

The purpose of the current pilot study is to evaluate whether it is feasible to conduct a clinical trial investigating CCBR for adults with cLBP in preparation for a subsequent, adequately powered randomized controlled trial (RCT) [98–101]. The primary objective is to test the feasibility and acceptability of 5 days of CCBR practice delivered via standardized audio recordings, compared to a structurally equivalent deep breathing sham control intervention [102–104], in a sample of 24 adults with cLBP. We expect it to be feasible to recruit, retain, and randomize participants to the intervention groups and expect at least 70% of participants to rate the interventions as acceptable and satisfying (≥ 7 out of 10). The secondary objective is to gather data regarding the plausibility that the CCBR intervention can result in clinically meaningful improvements in pain and pain-related variables [100, 105]. To demonstrate plausibility of improvement, we expect a greater ratio of participants in the CCBR group, compared to the control group, will meet the clinically significant treatment response target of ≥ 30% improvement in pre-post average pain intensity ratings [106–108]. A 30% reduction in pain is considered a moderately important change following treatment and is commonly used to determine clinical significance and/or treatment response in pain RCTs [107–111]. Results from this study will not be used to make claims about preliminary efficacy or to determine power for future sample size calculations [98, 100, 112] but rather to provide feasibility estimates, refine the interventions, and inform the successful design and implementation of an anticipated RCT, which will rigorously test the efficacy and mechanisms of this self-management breathing practice for adults with cLBP.

## Methods

### Study design

**Fig. 1:**
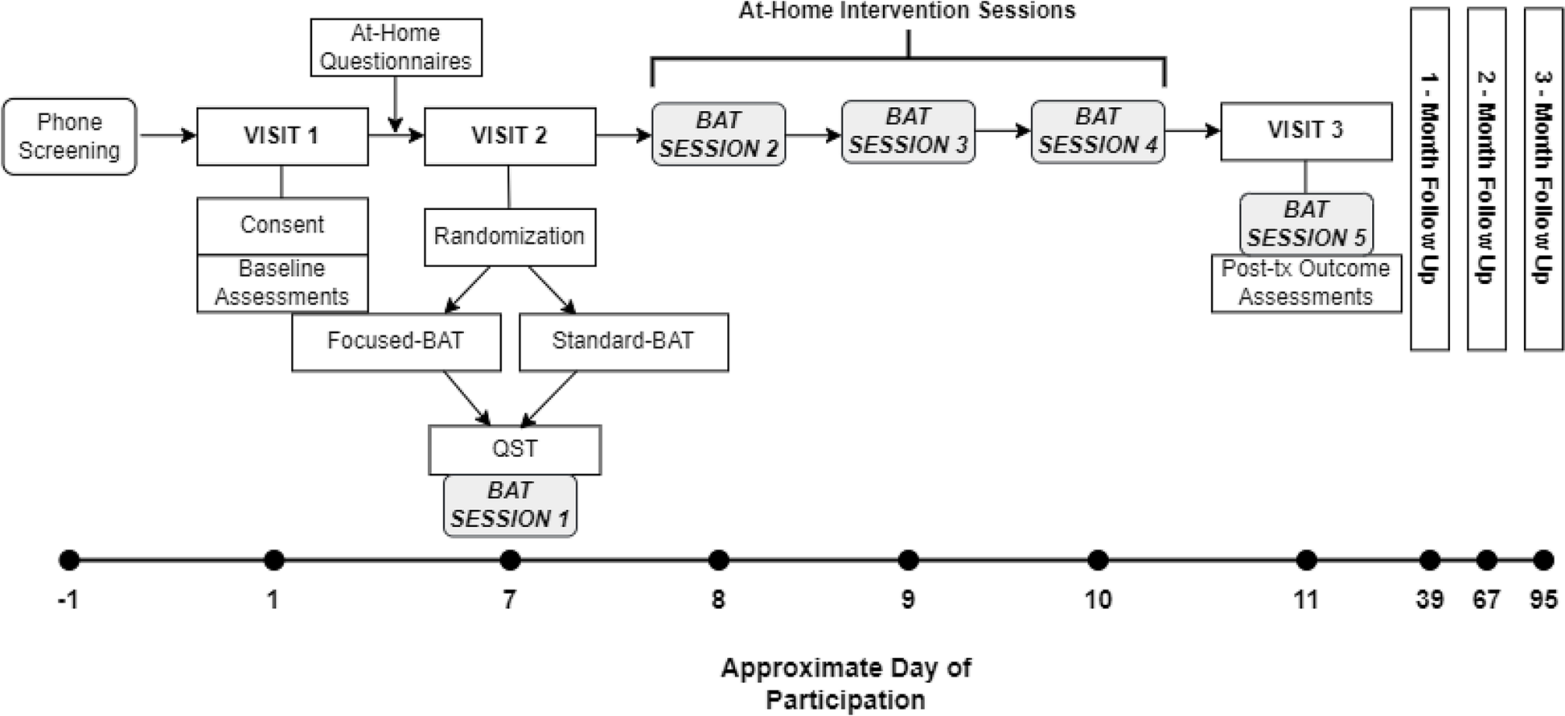
Overview of study design

This parallel group, pilot RCT is designed to examine the feasibility and acceptability of a 5-day conscious connected breathing intervention that includes brief periods of breath retention, compared to a deep breathing sham control intervention, in a sample of adults with cLBP. The intervention length (5 sessions, 17-min per session) was chosen because previous research has demonstrated that mind-body interventions can influence physiological mechanisms of interest, such as central and autonomic nervous system functioning, after a similar period of training [113]. Both interventions will be described as Breathing and Attention Training and neither will be depicted as the active therapeutic intervention (i.e., single-blind) in order to reduce possible expectancy and placebo effects common in pain research [102, 104, 114]. Participants will be randomly assigned at a 1:1 ratio to either the Standard-Breathing and Attention Training or the Focused-Breathing and Attention Training (BAT). The Standard-BAT is the sham control intervention, and the Focused-BAT is the active CCBR intervention. All participants will provide written and signed informed consent. The study was approved by the University of Florida Institutional Review Board, registered on clinicaltrials.gov (NCT04740710), and designed in accordance with CONSORT guidelines for reporting pilot RCTs [101, 115–117]. See Fig. 1 below for an overview of the study design.

### Procedure

Interested participants will complete a telephone screening to determine initial eligibility, and those who qualify will be scheduled for their first visit at the University of Florida Pain Clinical Research Unit to obtain informed consent and complete several baseline assessments and physical functioning tasks. At the end of Visit 1, participants will be scheduled for two additional study visits to occur within a 1-week period (pre-intervention and post-intervention). Participants will also be asked to complete two additional baseline surveys online using REDCap between Visit 1 and Visit 2, which is intended to reduce the participant burden and length of Visit 1. At Visit 2 (pre-intervention), participants will be randomized to the Standard- or Focused-BAT intervention before undergoing quantitative sensory testing procedures. Then, participants will be instructed via an audio-recording to practice their assigned breathing intervention for the first time with the researcher. Physiological data will be collected before, during, and after the breathing practice at both Visit 2 and Visit 3. Following Visit 2, participants will receive an email to practice BAT first thing in the morning if possible by following along to the recording on their own at home. If not possible to practice upon awakening, participants will be asked to practice at least 2 h after a meal. We will attempt to schedule intervention sessions over five consecutive days when possible (Monday–Friday). When not possible, we will require that the last at-home session and last in-person session (BAT sessions 4 and 5) be practiced on consecutive days. At Visit 3, participants will practice their assigned breathing intervention for the 5th and final time and then complete post-intervention questionnaires, quantitative sensory testing procedures, and physical functioning tasks. Finally, participants will be asked to complete 1-, 2-, and 3-month follow-up surveys online using REDCap.

### Participants

With an estimated attrition rate of 20%, we are recruiting 30 participants between the ages of 18 and 65 with cLBP from the Gainesville, FL community to obtain complete data on 24 participants. We anticipate an additional 75 participants who undergo screening procedures will be ineligible for the study. The sample size was chosen to be large enough to achieve the primary objective (i.e., evaluating feasibility and acceptability) while also considering practical constraints, such as funding and time. Therefore, no power analysis was conducted. Participants will be compensated up to $245 for completing all study procedures. Inclusion and exclusion criteria are displayed in Table 1 and are consistent with definitions for chronic primary low back pain (e.g., back pain associated with emotional distress and/or functional disability and not better accounted for by another condition) [118] and recommendations of the NIH Task Force on Research Standards for cLBP (i.e., participants with cLBP of at least moderate impact that has persisted on more days than not over the past 6 months) [119].

**Table 1:** Study inclusion and exclusion criteria

Inclusion criteria	
1. 18–65 years of age	
2. Low back pain that has persisted for at least 3 months and causes pain on most days (more days than not) over the past 6 months.	
3. Average pain intensity ≥ 4 out of 10 for the past week.	
Exclusion criteria	
1. Diagnosis of a systemic rheumatic disease or condition (e.g., rheumatoid arthritis, systemic lupus erythematosus, fibromyalgia).	
2. Asthma, breathing problems, or a respiratory disorder (e.g., chronic obstructive pulmonary disease).	
3. Daily use of opioids.	
4. Uncontrolled hypertension (i.e., systolic blood pressure/diastolic blood pressure > 150/95), orthostatic hypotension (e.g., issues with fainting), or cardiovascular or peripheral arterial disease.	
5. Current or past diagnosis of a neurological disease (e.g., Parkinson’s, multiple sclerosis, epilepsy, vasovagal syncope) or evidence of previous brain injury, including stroke and traumatic brain injury.	
6. Serious psychiatric disorder requiring hospitalization within the past 12 months.	
7. Current substance use disorder or history of hospitalization for treatment of a substance use disorder.	
8. Current participation in another research study involving an intervention or treatment.	
9. Currently pregnant	
10. Any significant comorbidities or issues that, in the opinion of the investigators, could interfere with the study or lead to deleterious effects for the participant.	

### Recruitment, screening, and enrollment

We will recruit for a “Breathe for Pain Study” widely throughout the Gainesville and University of Florida (UF) community to obtain a sufficient sample of eligible adults with cLBP. Recruitment flyers will be posted throughout the UF Campus (e.g., bulletin boards), as well as at local businesses (e.g., grocery stores), clinical settings (e.g., Clinical Research Center, Orthopedic Institute), and other locations recommended by the UF Clinical and Translational Science Institute Recruitment Center. The recruitment flyer and study information will also be posted online in relevant locations (e.g., The Pain Research and Intervention Center of Excellence Study Listings page). We will also call participants from UF registries, including HealthStreet a community engagement program that connects local residents with relevant research studies.

Potential participants will be screened over the phone using a standardized script. The phone screening involves briefly explaining the purpose of the study, collecting basic demographic and contact information, and determining initial eligibility. Interested and eligible participants will then schedule their first study visit where they will sign the informed consent form in person. Trained study staff will enter responses to the screening questions into a secure REDCap database.

### Randomization and blinding

The principal investigator (S.P) generated the allocation sequence with a block size of two using an online random number generator (Random.org). Based on the allocation sequence, participants will be randomized (1:1 ratio) using the REDCap Randomization Module at the beginning of Visit 2 to either the Focused- or Standard-BAT. Participants will not be told which intervention they were randomized to until the end of the study, after the 3-month follow-up survey. Due to the inherent difficulties of blinding behavioral interventions, only the participant—not the researcher—will be blind to intervention assignment [102, 114, 120]. Effectiveness of participant blinding will be reported.

### Interventions

#### Breathing and Attention Training (BAT)

Participants are informed that they will be randomly assigned to either the Focused-Breathing and Attention Training or the Standard-Breathing and Attention Training. The consent form provides information that the Standard-BAT includes instruction on deep breathing and relaxation, and the Focused-BAT is similar to the Standard-BAT in most ways but includes extra instructions to help you focus and alter your breathing patterns. Thereafter, each intervention is referred to as BAT more generally to all participants in an attempt to evenly manage participant expectations and reduce potential placebo effects [102, 103, 121]. When first introducing the interventions to participants during Visit 2, they will receive the same information on the possible effects of BAT (see Additional file 1: Appendix 1).

Both interventions involve five separate, 17-min practice sessions of BAT. The first 12 min of both interventions include instructions to breathe in ways consistent with their assigned intervention, and both interventions include 5 min of silence at the end where participants are instructed to simply relax and lay still without modifying their breathing in any way. The interventions will be delivered via an audio recording in order to standardize the interventions across sessions and participants, thereby enhancing treatment fidelity through consistent intervention delivery. When practicing in-person, participants will remain reclined in a chair; when practicing at-home, participants will be instructed to lie down in a safe and comfortable position. Aside from the specific breathing instructions during the 12-min of BAT, all other aspects (e.g., setting, frequency, posture, facilitator) of the interventions will be identical [103, 104]. The scripts for each intervention are included in Additional file 1: Appendix 1.

#### Focused-BAT

Each session of the Focused-BAT intervention involves three rounds of conscious connected breathing and breath retention. For the breathing phase, participants are instructed to take about 40 deep connected breaths (no pause between inhale and exhale) at a rate of about 20 breaths per minute. The inhalation is encouraged to be deep into the abdomen (i.e., full breath in), and the exhalation is encouraged to be relaxed (i.e., let the breath go). For the breath retention phase, participants are instructed to hold their breath after the 40th *exhale*. The duration of the breath retention is at the discretion of the participant, but the audio recording prompts participants to inhale after approximately 1-, 1.5-, and 2-min for rounds 1–3, respectively, increasing the time of the breath hold each round. Although previous studies on this breathing practice have shown that breath retention was safe up to 3.5 min [47, 48], participants are clearly instructed to inhale when they feel an urge to breathe without forcing it or pushing beyond their limits (i.e., “just inhale when you need”). When the participant inhales to end the breath retention they are instructed to hold their breath again for 10–15 s. This process of deep connected breathing, extended breath retention after an exhale, and brief breath retention after an inhale is considered one round—participants practice three consecutive rounds of this procedure in a single session. In the first Focused-BAT session, the participant practices one round with the researcher to ensure understanding of the instructions before proceeding with the full three rounds. The guided audio recording includes inhale and exhale sounds to pace the breathing during the breathing phase and instructs participants to relax any areas of tightness, tension, or holding and to stay present and pay attention to their body and physical sensations during the breath retention phase (i.e., mindful body awareness).

#### Standard-BAT

Participants are told that the point of the Standard-BAT practice is to remain in an alert and attentive yet relaxed state by taking deep breaths every minute or so [122–124]. The recording first instructs participants to breathe in deeply through their nose for about 5 s and exhale slowly for about 5 s and to continue with this deep breathing on their own for the next minute. Then participants are told to allow their breathing to return to its normal and natural rhythm without trying to change it in anyway, remembering to take a few deep breaths every minute or so. About every minute or two, the audio recording prompts participants to take a couple of deep breaths and includes audible breathing sounds to pace the inhale and exhale. This intervention is designed to be similar in most aspects to the CCBR intervention except with different instructions on how to breathe or how to attend to one’s breathing. Thus, this group can truthfully be told that they have been randomly assigned to a breathing and attention training intervention without actually receiving instructions to bring attention to their body or alter their breathing pattern in a way similar to the CCBR group (i.e., connected breathing, breath holding, mindful body awareness). Previous research has shown this type of breathing and attention intervention decreases pain intensity and pain unpleasantness, but to a lesser degree than a comparable mindfulness meditation training [124] and slow breathing intervention [38]. This Standard-BAT is also expected to be less potent than other deep breathing interventions because there are multiple periods of silence and few instructions on how to breathe, which is in contrast to other interventions that constantly cue participants to breathe deeply and rhythmically [27, 125]. Although some participants may maintain deep breathing on their own, the intervention is intended to induce natural, relaxed, spontaneous breathing.

#### Intervention adherence

At-home intervention adherence will be monitored by collecting data that tracks the length of time a participant stays on the intervention page of the survey that includes the guided audio recording. Specifically, we will assume the intervention was adhered to if a participant stays on the intervention page of the survey for at least 10 min.

### Assessments and measures

#### Primary feasibility and acceptability outcomes

Feasibility and treatment acceptability are the primary outcomes of the proposed pilot study. Feasibility will be assessed by quantifying rates of participant recruitment, participant adherence, participant retention, and study assessments completion. Treatment acceptability and participant satisfaction will be assessed with several face-valid questions (e.g., How acceptable did you find this BAT treatment? How satisfied are you with this BAT treatment?) rated on a 11-point numerical rating scale (NRS) with anchors that match the content of each question (e.g., 0 = not at all acceptable, 10 = extremely acceptable). These assessments are described further in Additional file 1: Appendix 2 and are similar to those commonly used in other pilot feasibility studies to evaluate the acceptability of an intervention and feasibility of implementing the study protocol [126–131]. In order to capture other aspects of feasibility and acceptability, we will ask participants to answer several open-ended questions about their involvement with the intervention and study procedures more generally (e.g., Were there challenges to participating in this study for you?). No formal qualitative analyses will be conducted, but we will review participant responses to identify possible areas of improvement to the interventions or study procedures [131]. Safety and adverse events will also be recorded after each intervention session using a yes/no symptom checklist and an open-ended prompt for participants to disclose any other possible adverse effects.

#### Baseline assessment and secondary outcomes

Consistent with the purpose of pilot RCTs [100, 117], the additional measures reflect baseline, outcome, and possible process variables that we anticipate will be included in a subsequent efficacy trial [132]. Table 2 indicates the schedule for collecting these psychometrically sound measures, and Additional file 1: Appendix 2 includes more detailed descriptions. The baseline assessment will include demographic and pain and health history questions that align with items recommended by the NIH Task Force on Research Standards for cLBP and the Back Pain Consortium (BACPAC) minimum dataset [119]. Moreover, many of the biobehavioral and patient-reported measures of this study were selected to be consistent with BACPAC recommendations to (1) assess meaningful outcomes following an intervention and (2) include phenotyping measures that may predict who responds to an intervention [158]. Although considered secondary outcomes in the current study, the primary clinical pain outcome measures for a future efficacy trial will be pain intensity and pain interference. The NIH PROMIS pain intensity-short form scale [133] will be used in analyses to determine clinical significance for the proposed study and subsequent efficacy trial by assessing improvements over the last 7 days on average using an 11-point NRS (0 = no pain, 10 = most pain imaginable). Finally, participants will also complete behavioral and physiological assessments at pre- and post-intervention, including physical functioning tasks, quantitative sensory testing, and heart rate variability.

**Table 2:** Timeline of study assessments and measures

*Timepoint*	Baseline		Pre-intervention	Post-intervention	Follow-ups		
Variable, category, or measure	Screening	Visit 1	Visit 2	Visit 3	1 months	2 months	3 months
Demographic information	X	X					
Contact information	X						
Eligibility criteria	X	X					
Health history	X	X					
Pain history [119]	X	X					
NIH PROMIS Positive Affect and Well-being [133]		X					
Stress and Adversity Inventory^a^ [134]		X					
Feasibility and acceptability							
Treatment acceptability				X			
Treatment expectations [135]			X				
Blinding effectiveness				X			
Adverse events			X	X			
Secondary outcomes and process measures							
NIH PROMIS-Short forms (pain intensity, pain interference, depression, sleep disturbance, sleep-related impairment) [133, 136–138]		X		X	X	X	X
Brief Pain Inventory-Short form [139, 140]		X		X	X	X	X
Michigan Body Map [141, 142]		X					
Patients’ Global Impression of Change [143–145]				X	X	X	X
Oswestry Disability Index [146]		X		X	X		X
Pain Catastrophizing Scale [147]		X		X	X		X
Pain Self-Efficacy Questionnaire [148]		X		X	X		X
Perceived Stress Scale [149]		X		X	X	X	X
Generalized Anxiety Disorder 2-item [150]		X		X	X	X	X
Multidimensional Assessment of Interoceptive Awareness-2 [151]		X		X	X		X
Multidimensional Psychological Flexibility Inventory^a^ [152, 153]		X		X	X		X
Physical and physiological assessments							
Back performance scale [154]		X		X			
Short performance physical battery [155]		X		X			
Quantitative sensory testing [156, 157]			X	X			
Heart rate variability			X	X			
Oxygen saturation			X	X			
Blood pressure		X	X	X			

^a^Assessment will be completed online between Visit 1 and Visit 2

*NIH* National Institutes of Health, *PROMIS* Patient-Reported Outcomes Measurement Information System

#### Physical functioning assessments

Two tests will be used to assess physical functioning—the Short Physical Performance Battery (SPPB) [155] and the Back Performance Scale (BPS) [154]. The SPPB is a widely used measure of physical function and consists of three tasks: usual gait speed over 4 meters, standing balance, and a chair stand test. The BPS will be used to assess both functional performance and movement-evoked pain through five tasks that are considered difficult for people with cLBP [159, 160]. An assessor will rate physical functioning for participants on each task using a 0–3 scale (total score = 0–15 where higher scores represent worse physical functioning). To measure movement-evoked pain, participants are asked to rate their pain immediately after each task on a 101-point visual analogue scale where 0 = no pain and 100 = most intense pain imaginable.

#### Quantitative Sensory Testing (QST)

We will use QST to determine pain modulatory balance via pressure pain threshold (PPT), temporal summation (TS) of mechanical pain, and conditioned pain modulation (CPM). First, to determine PPT, a handheld pressure algometer with a 1-cm^2^ tip (Medoc, Ltd., AlgoMed, Ramat Yishai, Israel) will be applied to the participants trapezius muscle at a steadily increasing rate of pressure. The participant will press a button when the pressure sensation first becomes painful. This procedure will be repeated five times on the same trapezius muscle, and the mean pressure rating across the five trials will be used for analyses on remote pain sensitivity. Second, to determine remote and local TS of mechanical pain, we will use a 300 *g* nylon monofilament (Touchtest Sensory Evaluator 6.65) to deliver a pinprick sensation to the back of the hand (remote) and lumbar spine (local). A single pinprick stimulus will be delivered and the participant will give a verbal pain rating from 0 (no pain) to 100 (most intense pain imaginable). Then, a series of 10 stimuli (once per second) will be delivered to the same location, and the participant will again give a rating of the greatest pain intensity experienced during the 10 stimuli. This procedure will be repeated twice at each site (back of each hand and bilaterally on lumbar spine) and the trials for each site will be averaged. TS will be used as an indicator of pain facilitation and calculated for each site by subtracting the average rating of the single stimulus from the 10 repeated stimuli. Third, to determine CPM, the test stimulus will be PPT at the trapezius, as described above, and the conditioning stimulus will be immersion of the hand (contralateral to the PPT) in cold water for 1 min at 12 °C (ARCTIC A25 refrigerated bath with an SC150 immersion circulator; ThermoFisher Scientific, USA). PPT will be assessed immediately before cold water immersion, 30 s after immersion, and immediately after withdrawing the hand from immersion. CPM will be used as an indicator of pain inhibition and will be calculated by subtracting the PPT before immersion from the PPT during immersion.

#### Physiological assessments

Physiological data will be recorded throughout the first and last BAT session and summarized across three epochs: 5-min before BAT (resting baseline), 12 min during BAT (active breathing), and 5 min after the BAT practice (recovery) [161]. A Biopac MP150 system with BioNomadix transmitter and RSPEC-R and Oxy100E modules (MP150-BIOPAC Systems Inc., Goleta, CA, USA) will be used for data acquisition, and Biopac’s Acknowledge software will be used for data recording and analyses. We will use a 3-lead EEG, lead II configuration, to record HRV in both the frequency and time domains. A pulse oximeter on the finger of the participant will be used to measure oxygen saturation. To gather baseline data, participants will be asked to lay still for 5 min while reclined before beginning the intervention. Physiological data acquisition will continue throughout the 12-min BAT intervention, and the last 5 min of silence in each intervention will be considered post-test data. Blood pressure will also be measured while the participant is reclined with three continuous readings immediately before the baseline period and after the 5 min of silence. The following physiological parameters will be summarized for each intervention, separated by baseline, during BAT, and post-BAT: Low-frequency (LF), high-frequency (HF), low-to-high frequency (LF/HF), standard deviation of normal-to-normal intervals (SDNN), root mean square of successive differences between heartbeats (RMSSD), oxygen saturation, and blood pressure. We will attempt to schedule Visit 2 and 3 at the same time of day to provide the best comparison of pre-post physiological measures [161, 162].

### Statistical analyses

We will assess intervention group equivalence on baseline and outcome variables using relevant univariate tests (chi-square and Kruskal-Wallis) with statistical significance set at *p* < 0.05. Descriptive statistics (percentages, frequencies, means, standard deviations, and 95% confidence intervals) will be used to summarize the results at each timepoint and determine feasibility and acceptability. For feasibility, successful retention is defined as at least 70% of enrolled participants completing the intervention sessions and monthly follow-ups. Acceptability is defined as at least 70% of participants reporting high average ratings (≥ 7 of 10) on acceptability and satisfaction measures. In order to address the secondary objective examining the plausibility of clinical improvement, we will calculate the proportion of participants who report a “moderately important” pain reduction of 30% or greater for each intervention group using the ratings of average pain intensity over the past week. We also plan to explore potential covariates and predictors of treatment response (e.g., age, sex, QST).

## Discussion

This will be the first study to examine the effects of a conscious connected breathing with breath retention intervention for individuals with chronic pain. The successful completion of this early stage study will provide data regarding the feasibility and acceptability to conduct a future RCT testing the efficacy and mechanisms of this breathing self-management practice for adults with cLBP [131, 163]. The study is designed to be consistent with recommendations for conducting pilot RCTs [100, 117, 164, 165] and for intervention development [61, 166, 167]. Specifically, this pilot feasibility study is considered a smaller-scale version of an anticipated RCT where the target population is recruited and participants are randomly assigned to the active intervention or sham control intervention and complete all questionnaires, behavioral assessments, and procedures as planned in a future efficacy trial. Moreover, in accordance with models of intervention development, information gathered from this study will be used to refine the procedures, optimize the interventions, and adjust the elements of study design and implementation prior to proceeding with a larger trial. Given the need for safe, effective, and accessible treatments for chronic pain [5, 12, 16, 168] and the increasing usage of complementary and integrative health approaches [13], the proposed intervention focused on the power of conscious breathing for pain management has high potential impact as a standalone practice or adjunct to existing treatments [24, 25, 169].

There is currently little research examining the effects of breathing practices in chronic pain populations [23, 24, 170]. Although there is solid theoretical rationale to expect breathing practices and breathwork interventions to be beneficial for individuals suffering with chronic pain [22, 26, 33, 72, 85, 171], high-quality evidence for their efficacy is sparse [23–25]. The relatively few RCTs available reveal that various breathing interventions can improve back pain and pain-related outcomes, but significant heterogeneity in study design, quality, length of follow-ups, and type of breathing interventions limit any strong conclusions [23–25]. Nevertheless, we elected to test this particular conscious connected breathing intervention that also includes breath retention for several reasons. (1) There is empirical evidence supporting that this specific breathing practice influences biological activity relevant to chronic pain, such as decreased inflammation and adaptive immune, nervous, and metabolic activity [47–49, 58]. (2) This breathing technique is relatively easy to learn and quick to practice on one’s own [56]. Therefore, it is advantageous as a remote self-management practice that is expected to be more potent than simply deep breathing and more accessible compared to other emotionally activating breathwork interventions that need to be practiced in a specific setting under the supervision of a trained therapist or facilitator [42–45, 172–175]. (3) Millions of people around the world already engage in this breathing practice on their own^2^, but comparably little scientific research has tested its safety and efficacy. The proposed study is a precursor to a larger efficacy trial which is intended to help bridge this disconnect between widespread public usage and scientific evidence [5, 61]. (4) Finally, because breathing is free and accessible to everyone and the intervention can be practiced remotely with fidelity through the use of replicable audio, video, or app-based media, it has enormous potential for scalability as a self-management strategy.

The sham control intervention chosen for this early stage study deserves discussion. The purpose of the Standard-BAT condition is to minimize threats to internal validity by controlling for treatment non-specific factors (e.g., attention, expectations) and key treatment specific factors (e.g., breathing instructions, relaxation) [52, 53, 102, 176–179]. Holding most components of the BAT interventions constant addresses the well-known issue of placebo effects in pain research [102–104, 180–183] and enhances internal validity of the study by isolating the putative therapeutic mechanism(s) of the Focused-BAT/CCBR intervention (i.e., connected breathing with retention technique, mindful body awareness, and consciously releasing tension). While we do not expect the Standard-BAT to be completely inert, as demonstrated by previous studies showing a similar sham intervention reduces pain sensitivity and unpleasantness [38, 124], it is expected to be less effective than CCBR, yet equivalent in terms of engagement, credibility, and expectations of benefit. As such, the Standard-BAT is considered a stringent or highly formidable control condition. The use of a high formidability control condition, compared to a low formidability control condition (e.g., waitlist), increases the chances of type II error, or the incorrect inference that there is no difference between the treatment arms when there indeed is [52, 53, 104, 176]. Therefore, some researchers recommend against a highly formidable control group in early stage studies because small treatment effects may lead to prematurely abandoning the development of a promising intervention [52, 184]. Nevertheless, the current study design aligns with the purpose of pilot RCTs in that it will provide feasibility data regarding participants’ satisfaction, expectations, and adherence to the interventions that are anticipated to be used in a larger trial. Moreover, this pilot study will provide valuable data regarding the acceptability and credibility of this control condition. While no control condition is perfect, the Standard-BAT in this study is structurally equivalent to the Focused-BAT with respect to treatment format, implementation, and several other treatment specific and non-specific factors, which will allow for strong causal conclusions regarding treatment effects and mechanisms in future studies [102–104].

Although there are several strengths to the proposed study, such as the multimethod assessments, standardized interventions, and a randomized controlled design, there are limitations to highlight. Considering this pilot RCT is a smaller version of a future efficacy trial, the current “dose” of a 5-day CCBR practice may be insufficient for clinically meaningful changes in the proposed primary efficacy outcomes of pain intensity and pain interference. It may be that this self-management practice needs to be maintained over a longer period of time for substantial or sustained benefits. For example, clinical trials of breathing exercises typically involve daily practice for at least 4 weeks and often up to 8 or 12 weeks [24, 25, 185]. Moreover, while we can visually monitor treatment fidelity during the in-person BAT sessions, we will be unable to determine fidelity when participants practice the interventions at-home. Additionally, there may be unknown safety issues with the CCBR intervention (e.g., cyclic hyperventilation, breath holding, mindful body awareness) that are over- or under-represented in the small sample size of the current study. We will monitor adverse events by soliciting expected and unexpected side-effects of the intervention, but, ultimately, a larger study is needed to establish the safety of the intervention. Finally, we caution overinterpretation of the results regarding the plausibility of improvement in pain-related outcomes. These analyses are considered exploratory to discern whether the intervention has any effects at all by providing initial evidence that the target of clinical significance (≥ 30% improvement in pain intensity), rather than statistical significance, is achievable for some people [100, 105, 106, 132]. If the ratio of participants showing meaningful improvement favors the control, we may need to modify the sham control intervention or consider a three-arm trial design for the larger follow-up trial [182].

In conclusion, this study is the first step to advance a line of research testing whether this novel, innovative intervention is safe and effective as a self-management practice for those with cLBP. The proposed intervention empowers people to care for themselves by taking advantage of their breathing and attention to self-regulate their body and mind. Subsequent efficacy testing of this breathing practice, if feasible, has high potential to advance our understanding of pain mechanisms and identify a promising new self-management intervention for chronic pain.

## Supplementary Information

**Additional file 1 **: **Appendix 1**. Breathing and Attention Training Description to Participants. **Appendix 2**. Assessments and Measures.

## Data Availability

When the study is completed, de-identified data will be made available from the corresponding author on reasonable request.

## Abbreviations

cLBP: Chronic low back pain
CCBR: Conscious connected breathing with breath retention
HRV: Heart rate variability
RCT: Randomized controlled trial
BAT: Breathing and Attention Training
UF: University of Florida

## Footnotes

1 The connected breathing with breath retention intervention is commonly known as the Wim Hof Method or Wim Hof Breathing because it was popularized by a man named Wim Hof. Much of the early research on this intervention include either Wim Hof as a case study or participants who were personally trained by him.

2 As of 1/14/2022, there are over 39 million views on the top Wim Hof Breathing video on Youtube (“Guided Wim Hof Breathing Method”) and many other similar guided breathing videos have several million views.
