# Supplementary material for: Conscious connected breathing with breath retention intervention in adults with chronic low back pain: protocol for a randomized controlled pilot study
Source: Pilot Feasibility Stud. 2023 Jan 24;9:15. doi: 10.1186/s40814-023-01247-9 (PMC9872326; doi:10.1186/s40814-023-01247-9)
Supplement: Supplementary file 1 — Additional file 1 : Appendix 1. Breathing and Attention Training Description to Participants. Appendix 2. Assessments and Measures. [file 40814_2023_1247_MOESM1_ESM.docx]

**Appendix A**

**Breathing and Attention Training Description to Participants**

When first introducing the interventions to participants during Visit 2, they will receive the same information on the possible effects of BAT. Specifically, they will be told: “This type of deep breathing changes our body’s chemistry. So, you may experience symptoms of tingling, lightheadedness, change in body temperature (hot or cold), looseness in the body, and cramping, but that’s all normal and OK. It’s from changing our physiology and the carbon dioxide and oxygen levels in the body. When you feel these things just keep breathing and know that you are safe and these effects are normal. And remember, you can choose to stop the breathing intervention at any time.”

**Focused-BAT Script**

0:00 – Make sure you are lying down in a safe and comfortable position.

0:06 – Allow your body to relax.

0:09 – Now, let’s begin round 1.

0:12 – Full breath in, let it go. Full breath in, let it go. *(Inhale and exhale noises begin).*

0:19 – Inhale, exhale. Inhale, exhale.

0:25 – Remember, it’s a circular breath with no pause between inhalation and exhalation.

0:31 – Inhale, exhale. Inhale, exhale.

0:37 – Fully into your belly, your chest, and out. Fully in, letting go. Fully in, letting go. Fully in, letting go.

0:51 – Keep breathing. Full breath in, and out. Not fully out where you’re pushing, but fully in.

1:14 – Full breath in, let it go. Full breath in, let it go. Full breath in, let it go. Follow along to the sounds of the breath.

1:33 – 10 more.

1:37 – Deeply in, letting go. Deeply in, letting go.

1:46 – Fully in, letting go. Fully in, letting go.

2:11 – Last one. Full breath in and let it go and hold your breath. *(Breath retention begins).*

2:18 – Just relax. Stay here in the present moment. And notice the sensations in your body.

2: 29 – Let the relaxation spread across your whole body.

2: 35 – If you notice you are holding, tensing, or tightening any areas of your body, see if you can relax there and let it go.

2: 48 – *(30 seconds in).* When you feel the urge to breath, it’s ok. You can inhale when you need to. You don’t need to force it or push it beyond your limits. Just inhale when you need.

3: 02 – We’ll take a recovery breath in 5, 4, 3, 2, 1.

3:11 – Deep inhale and hold it for 10-15 seconds. *(Breath retention for up to 1 minute begins for Round 1).*

3:21 – Exhale in 3, 2, 1. And that’s the end of Round 1.

3:30 – Now, let’s begin Round number 2.

3: 33 – Full breath in, let it go. Full breath in, let it go. Inhale, exhale. Inhale, exhale. Remember it’s a circular breath with no pause between inhalation and exhalation. Inhale, exhale, inhale exhale, inhale exhale.

3:59 – Fully into your belly, your chest and out. Fully in, letting go. Fully in, letting go. Fully in, letting go. Keep breathing.

4:15 – Full breath in, and out. Not fully out where you’re pushing, but fully in.

4: 35 – Full breath in, let it go. Full breath in, let it go. Full breath in, let it go. Follow along to the sounds of the breath.

4:54 – Ten more. Deeply in, letting go. Deeply in, letting go.

5: 07 – Fully in, letting go. Fully in, letting go.

5:32 – Last one, full breath in. And Let it go, and hold your breath.

5:37 – *(Breath retention begins for up to 1.5 minutes begins for Round 2).*

5:40 – Just relax, stay here in the present moment. Become aware of your body. Notice the sensations throughout your body. Let your body relax any areas of tension or tightness.

6:12 – Let the relaxation spread across your whole body.

6:32 – If you need to breathe before I give the cue, that’s ok. Whenever you feel the urge to breath, then breathe.

6:49 – Almost there.

6:55 – We’ll take a breath in 5-4-3-2-1.

7:01 – Deep inhale, and hold it.

7:12 – And exhale in 3-2-1. And that’s the end of Round 2.

7:21 – We’ll begin Round 3 now.

7:26 – Fully in, letting go. Fully in, letting go. Fully in, letting go. Follow along to the sounds of breathing.

7:54 – Fully in, letting go. Finding a rhythm of circular breathing.

8:04 – Inhale, exhale. Inhale, exhale. Inhale, exhale.

8:20 – Fully into the belly, chest, and letting go.

8:33 – Fully in, letting go. Fully in, letting go.

8:46 – Keep going.

8:55 – 10 more.

9:00 – Fully in, letting go. Fully in, letting go.

9:24 – Last one. Full breath in, and let it go, and hold. Holding your breath now. Just relax into this silence. *(Breath retention for up to 2 minutes begins for Round 3).*

9:58 – If you notice you’re holding, tensing, or tightening any areas of your body, see if you can relax there and let it go. Let the relaxation spread across your whole body.

10:55 – Remember if you need to breathe before I give the cue, it’s ok to inhale. Just whenever you feel the urge to breathe, then you breathe.

11: 09 – We’re almost there.

11:20 – Take a breath in 5-4-3-2-1.

11:26 – Deep inhale and hold for 10-15 seconds.

11:37 – Exhale in 3-2-1. And that’s the end of round number 3.

11:44 – Now, just let your breathing return to normal without trying to change it in any way. Stay present and aware of the sensations in your body as you continue to relax and lay here for the next 5 minutes.

17:01 – When you’re ready, you can gently open your eyes and come back to the room. Make any slight adjustments that you need before slowly, coming to sit up. And that’s the end of our practice today.

**Standard-BAT Script**

0:03 – Make sure you are lying down in a safe and comfortable position.

0:10 – Let your body relax.

0:13 – Let’s begin by breathing deeply in and out through the nose. Inhaling for about 5 seconds, pausing, and then exhaling for about 5 seconds. Again, inhale, pause, long exhale. Relaxing the body with the exhale.

0:42 – Continue with this deeper than normal breathing for the next minute or so.

*Silence for 1 minute from 0:42 to 1:49*

1:49 – The point of this practice is to take some deep breaths every minute or so. And try to maintain an alert and attentive yet relaxed state. Taking deep breaths every so often helps us stay in that relaxed wakefulness.

*Silence for 50 seconds from 2:03 to 2:57*

2:57 – Take a couple deep breaths. Inhaling deeply into the abdomen, and exhaling fully. *(2 audible breathing sounds)*. Now allow your breath to return to its normal and natural rhythm without trying to change it in any way, remembering to take a few deep breaths every minute or so.

*Silence for 2 minutes from Silence from 3:31 to 4:57*

4:57 – Audible breathing sounds. *(2 audible inhales and exhales).*

*Silence for 1 minute from 5:19 to 6:27*

6:27 Staying awake and alert, yet also relaxed. *(2 audible inhales and exhales).*

*Silence for 1 minute from 6:30 to 7:41.*

7:41 – Audible breathing sounds. *(2 audible inhales and exhales).*

*Silence for 1 minute from 8:02 to 9:20.*

9:20 – Remember we don’t want to fall asleep or get too relaxed. We’re looking for a balanced state of attention and relaxation. Right in the middle. *(2 audible inhales and exhales).*

*Silence for 2 minutes from 9:50 to 12:03.*

12:04 – Take a couple deep breaths as you lay here in silence for the last 5 minutes. *(2 audible inhales and exhales).*

17:01 – When you’re ready, you can gently open your eyes and come back to the room. Make any slight adjustments that you need before slowly, coming to sit up. And that’s the end of our practice today.

**Appendix B**

**Assessments and Measures**

***Primary Feasibility and Acceptability Outcomes***For participant recruitment, we will maintain a detailed record of the recruitment process, including the number of interested participants screened, the number of participants eligible after screening, and reasons for ineligibility. We will report the enrollment-to-screening ratio, which will include the number of participants enrolled out of the number of potential participants screened, and we will also report the proportion of eligible participants enrolled. Participant adherence rate will be reported as a percentage of intervention sessions completed. Retention feasibility will be assessed as a percentage of participants who complete at least 4 out of 5 intervention sessions and 2 out of 3 monthly follow-ups. Participants who drop out of study will be contacted to inquire about reasons for withdrawing. The feasibility of assessment procedures will be assessed as a percentage of scheduled outcome assessments completed in the given timeframe as an average from all participants who completed treatment.

Treatment acceptability and participant satisfaction is another primary outcome of the proposed study and will be assessed with several face-valid questions (e.g., How acceptable did you find this BAT treatment? How satisfied are you with this Breathing and Attention Training treatment?). Each item will be rated on a 11-point numerical rating scale (NRS) with anchors that match the content of each question (e.g., 0 = Not at all acceptable, 10 = Extremely acceptable). These items are similar to those commonly used in other pilot feasibility studies to evaluate the acceptability of an intervention and feasibility of implementing the study protocol (1-6). The items will be reported separately and averaged to create a single summary measure of treatment acceptability to be used in analyses. Two additional yes/no questions will inquire about participants’ interest in another trial using this same BAT intervention and whether they would recommend this BAT to a friend. Three additional questions will ask about participant’s perception of the length of the BAT practice. The questions and response formats are as follows: 1.) You just completed 5-days of BAT, how satisfied were you with the length of this treatment program (i.e., 5-days)? (-5 = Too short, 0 = Just right, 5 = Too long); 2.) The study you just completed had 5 days of BAT. If instead it involved 30 days of BAT, would you have been willing to practice for all 30 days? (yes or no); and 3) If yes, out of 30 days, how many days would you be willing to practice? (open-ended response with a number between 1-30 days). These questions will provide preliminary information regarding participant’s satisfaction with the current length of the intervention and willingness to engage in a longer intervention.

***Safety***

Blood pressure will be assessed before proceeding with pain testing procedures and the BAT interventions. If the average blood pressure across three consecutive readings is above 150 systolic or 95 diastolic, and remains above 150/95 after checking again five minutes later, then the study visit will be terminated and rescheduled. Potential adverse events will be recorded before and after all five intervention sessions by asking participants if they have experienced, are currently experiencing, or experienced during BAT any of the following effects: tingling, headache, lightheadedness/dizziness, visual sensations (e.g., seeing lights), or ringing in your ears. These items will be rated on an 11-point scale (0 = none or not at all, 10 = extremely). In an attempt to capture other possible adverse events, participants will be asked if they are experiencing any other effects that were not listed (yes/no) and given the opportunity to describe them in an open-ended prompt.

***Pain Intensity and Pain Interference***The primary clinical pain outcomes for a future efficacy trial will be pain intensity and pain interference. The PROMIS pain intensity-short form scale (7) will be used for primary analyses to assess pain intensity over the last 7 days on average using an 11-point scale (0 = No pain, 10 = Most pain imaginable). The PROMIS pain interference-short form scale (8) will assess pain interference over the past week on a 5-point scale (1 = Not at all, 5 = Very much) in the following domains: enjoyment of life, ability to concentrate, day-to-day activities, recreational activities, tasks away from home, and socializing with others. The Brief Pain Inventory-Short Form (BPI-SF) will also measure pain intensity and pain interference in slightly different ways to capture a more complete understanding of participants’ pain experience. Specifically, the BPI-SF will measure pain intensity over the last 24 hours on average, at its worst, as well as right now, on the same 11-point scale. Participants will also rate how much pain interfered with the following activities in the past 24 hours on a 0 (Does not interfere) to 10 (Completely interferes) scale: General activity, mood, walking ability, normal work, relations with other people, and sleep.

***Patients’ Global Impression of Change (PGIC)***This single-item measure asks participants to describe any changes in activity limitations, symptoms, emotions, and overall quality of life, related to your low back since the beginning of BAT. Participants will respond on a 7-point scale ranging from 1 (No change or condition has gotten worse) to 7 (A great deal better, and a considerable improvement that has made all the difference). This item aligns with IMMPACT (Initiative on Methods, Measurement, and Pain Assessment in Clinical Trials) recommendations for pain research (9) and is commonly used to evaluate clinically significant improvements following an intervention (10, 11).

***Treatment Expectations***
After participants practice the BAT intervention for the first time they will be asked to complete the 7-item Stanford Expectations of Treatment Scale (12). Participants will rate the extent to which they agree or disagree with how they think they will respond to the treatment using a 7-point scale (1 = Strongly disagree, 7 = Strongly agree). The scale measures positive treatment expectancies (e.g., “This treatment will be completely effective”) and negative treatment expectancies (e.g., “I am worried about this treatment”)—these two subscales will be calculated separately. Two additional items will assess treatment expectations: “How helpful do you expect the breathing and attention training (BAT) will be to reduce your current low back pain?” (0 = Not at all helpful, 10 = Extremely helpful), and “What is your best guess about the chance that you will be free of symptoms like pain, stiffness, numbness, weakness, etc. after your breathing and attention training treatment?” (No chance, Small chance, Moderate chance, Substantial chance, Certain (100%).

***Blinding Assessment***
At the post-intervention session, we will test for the adequacy of participant blinding. Specifically, we will assess participants’ accuracy of assigned treatment condition, certainty of treatment condition assignment, and an open-ended question as to their reasoning for which condition they believed they were in. The first question will state: “At the beginning of the study, we told you that you would either receive Standard-Breathing and Attention Training or Focused-Breathing and Attention Training. Which type of Breathing and Attention Training do you feel you received?”. The next question will “pipe” in their response from the previous question and ask “how certain are you about being in this condition” on a 7-point scale (1 = Not at all certain, 7 = Very certain). Finally, participants will respond to an open-ended question that asks why they think they were in the condition they chose.

***Michigan Body Map***
Along with the BPI-SF, participants will indicate on the Michigan Body Map (MBM) all of the areas on their body where they have felt chronic pain for the past 3 months (13, 14). The MBM is a general measure of widespread body pain, and the number of pain sites (up to 35) endorsed by participants will be summed and used as a potential indicator of central sensitization (15).

***Acute treatment effects***
Immediately before and after each intervention session, we will assess participants’ pain intensity, pain unpleasantness, pain interference, and mood. The pre-intervention questions will measure current pain and mood and past 24-hour levels of pain experiences using an 11-point NRS relevant to each item. The post-intervention questions will measure current pain, experience of the BAT session (-5 = Extremely unpleasant, 5 = Extremely pleasant), and the same mood items regarding feelings right now (e.g., How relaxed do you feel?, How much muscle tension or tightness do you feel right now?, How stressed do you feel?). Previous work shows the utility of these questions to demonstrate meaningful, short-term effects of an intervention (16).

**Psychosocial Assessments**
We will include several other reliable and valid psychosocial measures that may be included as secondary outcomes or process variables in a future efficacy trial. The 10-item Oswestry Disability Index (ODI) (17) assesses back pain-related disability across 10 domains (e.g., personal care, lifting, walking, sleeping). The 13-item Pain Catastrophizing Scale (PCS) (18) measures negative thought and feeling patterns associated with being in pain (e.g., “I worry all the time about whether the pain will end.”). The Pain Self-Efficacy Questionnaire (PSEQ) (19) includes 10 items to assess participants’ beliefs in their ability to function despite the pain. The 10-item Perceived Stress Scale (20) will assess participants’ subjective levels of stress over the past week. The 2-item Generalized Anxiety Disorder scale will assess feelings of anxiety and worrying over the past week (4, 21, 22). Several NIH PROMIS-short form measures (7, 8, 23, 24) will be used to assess depression, sleep disturbance, sleep-related impairment, and well-being. The Multidimensional Assessment of Interoceptive Awareness-2 (MAIA-2; (25) will be used as a self-report measure of interoception. The 24-item Multidimensional Psychological Flexibility Inventory-Short form (MPFI) (26) will be used to assess the six dimensions of psychological flexibility (e.g., acceptance, present moment awareness, cognitive defusion) and six dimensions of psychological inflexibility (e.g., experiential avoidance, cognitive fusion, inaction). Finally, we will include the Stress and Adversity Inventory (STRAIN), an online interview-based measure that assesses the presence and severity of a variety of possible challenging and stressful events that may have occurred over one’s lifespan (27, 28).

References

1. Darnall BD, Sturgeon JA, Kao MC, Hah JM, Mackey SC. From Catastrophizing to Recovery: a pilot study of a single-session treatment for pain catastrophizing. J Pain Res. 2014;7:219-26.

2. Lysne PE, Palit S, Morais CA, DeMonte LC, Lakdawala M, Sibille KT, et al. Adaptability and Resilience in Aging Adults (ARIAA): protocol for a pilot and feasibility study in chronic low back pain. Pilot Feasibility Stud. 2021;7(1):188.

3. Greenberg J, Popok PJ, Lin A, Kulich RJ, James P, Macklin EA, et al. A Mind-Body Physical Activity Program for Chronic Pain With or Without a Digital Monitoring Device: Proof-of-Concept Feasibility Randomized Controlled Trial. JMIR Form Res. 2020;4(6):e18703.

4. Sherman KJ, Wellman RD, Hawkes RJ, Phelan EA, Lee T, Turner JA. T'ai Chi for Chronic Low Back Pain in Older Adults: A Feasibility Trial. J Altern Complement Med. 2020;26(3):176-89.

5. Salwen-Deremer JK, Smith MT, Aschbrenner KA, Haskell HG, Speed BC, Siegel CA. A pilot feasibility trial of cognitive-behavioural therapy for insomnia in people with inflammatory bowel disease. BMJ Open Gastroenterol. 2021;8(1).

6. Aschbrenner KA, Kruse G, Gallo JJ, Plano Clark VL. Applying mixed methods to pilot feasibility studies to inform intervention trials. Pilot and feasibility studies. 2022;8(1):1-13.

7. Cella D, Riley W, Stone A, Rothrock N, Reeve B, Yount S, et al. The Patient-Reported Outcomes Measurement Information System (PROMIS) developed and tested its first wave of adult self-reported health outcome item banks: 2005–2008. Journal of clinical epidemiology. 2010;63(11):1179-94.

8. Amtmann D, Cook KF, Jensen MP, Chen W-H, Choi S, Revicki D, et al. Development of a PROMIS item bank to measure pain interference. Pain. 2010;150(1):173-82.

9. Dworkin RH, Turk DC, Wyrwich KW, Beaton D, Cleeland CS, Farrar JT, et al. Interpreting the clinical importance of treatment outcomes in chronic pain clinical trials: IMMPACT recommendations. The journal of pain. 2008;9(2):105-21.

10. Ferguson L, Scheman J. Patient global impression of change scores within the context of a chronic pain rehabilitation program. The Journal of Pain. 2009;10(4):S73.

11. Scott W, McCracken LM. Patients' impression of change following treatment for chronic pain: global, specific, a single dimension, or many? J Pain. 2015;16(6):518-26.

12. Younger J, Gandhi V, Hubbard E, Mackey S. Development of the Stanford Expectations of Treatment Scale (SETS): a tool for measuring patient outcome expectancy in clinical trials. Clinical Trials. 2012;9(6):767-76.

13. Brummett CM, Bakshi RR, Goesling J, Leung D, Moser SE, Zollars JW, et al. Preliminary validation of the Michigan Body Map. Pain. 2016;157(6):1205-12.

14. Hassett AL, Pierce J, Goesling J, Fritsch L, Bakshi RR, Kohns DJ, et al. Initial validation of the electronic form of the Michigan Body Map. Reg Anesth Pain Med. 2019.

15. McKernan LC, Johnson BN, Crofford LJ, Lumley MA, Bruehl S, Cheavens JS. Posttraumatic Stress Symptoms Mediate the Effects of Trauma Exposure on Clinical Indicators of Central Sensitization in Patients With Chronic Pain. Clin J Pain. 2019;35(5):385-93.

16. Feinstein JS, Khalsa SS, Yeh HW, Wohlrab C, Simmons WK, Stein MB, et al. Examining the short-term anxiolytic and antidepressant effect of Floatation-REST. PLoS One. 2018;13(2):e0190292.

17. Fairbank JC, Pynsent PB. The Oswestry disability index. Spine. 2000;25(22):2940-53.

18. Sullivan MJ, Bishop SR, Pivik J. The pain catastrophizing scale: development and validation. Psychological assessment. 1995;7(4):524.

19. Nicholas MK. The pain self-efficacy questionnaire: taking pain into account. European journal of pain. 2007;11(2):153-63.

20. Cohen S, Kamarck T, Mermelstein R. Perceived stress scale. Measuring stress: A guide for health and social scientists. 1994;10(2):1-2.

21. Skapinakis P. The 2-item Generalized Anxiety Disorder scale had high sensitivity and specificity for detecting GAD in primary care. 2007.

22. Cherkin DC, Sherman KJ, Balderson BH, Cook AJ, Anderson ML, Hawkes RJ, et al. Effect of mindfulness-based stress reduction vs cognitive behavioral therapy or usual care on back pain and functional limitations in adults with chronic low back pain: a randomized clinical trial. Jama. 2016;315(12):1240-9.

23. Yu L, Buysse DJ, Germain A, Moul DE, Stover A, Dodds NE, et al. Development of short forms from the PROMIS™ sleep disturbance and sleep-related impairment item banks. Behavioral sleep medicine. 2012;10(1):6-24.

24. Schalet BD, Pilkonis PA, Yu L, Dodds N, Johnston KL, Yount S, et al. Clinical validity of PROMIS Depression, Anxiety, and Anger across diverse clinical samples. J Clin Epidemiol. 2016;73:119-27.

25. Mehling WE, Acree M, Stewart A, Silas J, Jones A. The Multidimensional Assessment of Interoceptive Awareness, Version 2 (MAIA-2). PLoS One. 2018;13(12):e0208034.

26. Rolffs JL, Rogge RD, Wilson KG. Disentangling components of flexibility via the hexaflex model: Development and validation of the Multidimensional Psychological Flexibility Inventory (MPFI). Assessment. 2018;25(4):458-82.

27. Slavich GM. Life Stress and Health: A Review of Conceptual Issues and Recent Findings. Teach Psychol. 2016;43(4):346-55.

28. Slavich GM, Shields GS. Assessing Lifetime Stress Exposure Using the Stress and Adversity Inventory for Adults (Adult STRAIN): An Overview and Initial Validation. Psychosom Med. 2018;80(1):17-27.
